# Assessment of the Inclusion of Racial/Ethnic Minority, Female, and Older Individuals in Vaccine Clinical Trials

**DOI:** 10.1001/jamanetworkopen.2020.37640

**Published:** 2021-02-19

**Authors:** Laura E. Flores, Walter R. Frontera, Michele P. Andrasik, Carlos del Rio, Antonio Mondríguez-González, Stephanie A. Price, Elizabeth M. Krantz, Steven A. Pergam, Julie K. Silver

**Affiliations:** 1 College of Allied Health Professions, University of Nebraska Medical Center, Omaha; 2 Department of Physical Medicine, Rehabilitation and Sports Medicine and Department of Physiology, University of Puerto Rico School of Medicine, San Juan; 3 Vaccine and Infectious Disease Division, Fred Hutchinson Cancer Research Center, Seattle, Washington; 4 Department of Medicine, Emory University School of Medicine, Atlanta, Georgia; 5 Hubert Department of Global Health, Rollin School of Public Health of Emory University, Atlanta, Georgia; 6 Universidad Central del Caribe, Bayamón, Puerto Rico; 7 Hellen Keller International, New York, New York; 8 Department of Physical Medicine and Rehabilitation, Harvard Medical School, Boston, Massachusetts; 9 Spaulding Rehabilitation Hospital, Charlestown, Massachusetts; 10 Department of Physical Medicine and Rehabilitation, Massachusetts General Hospital, Boston; 11 Department of Physical Medicine and Rehabilitation, Brigham and Women's Hospital, Boston, Massachusetts

## Abstract

**Importance:**

Medical research has not equitably included members of racial/ethnic minority groups or female and older individuals. There are limited data on participant demographic characteristics in vaccine trials despite the importance of these data to current trials aimed at preventing coronavirus disease 2019.

**Objective:**

To investigate whether racial/ethnic minority groups and female and older adults are underrepresented among participants in vaccine clinical trials.

**Design, Setting, and Participants:**

This cross-sectional study examined data from completed US-based vaccine trials registered on ClinicalTrials.gov from July 1, 2011, through June 30, 2020. The terms *vaccine*, *vaccination*, *immunization*, and *inoculation* were used to identify trials. Only those addressing vaccine immunogenicity or efficacy of preventative vaccines were included.

**Main Outcomes and Measures:**

The numbers and percentages of racial/ethnic minority, female, and older individuals compared with US census data from 2011 and 2018. Secondary outcome measures were inclusion by trial phase and year of completion.

**Results:**

A total of 230 US-based trials with 219 555 participants were included in the study. Most trials were randomized (180 [78.3%]), included viral vaccinations (159 [69.1%]), and represented all trial phases. Every trial reported age and sex; 134 (58.3%) reported race and 79 (34.3%) reported ethnicity. Overall, among adult study participants, White individuals were overrepresented (77.9%; 95% CI, 77.4%-78.4%), and Black or African American individuals (10.6%; 95% CI, 10.2%-11.0%) and American Indian or Alaska Native individuals (0.4%; 95% CI, 0.3%-0.5%) were underrepresented compared with US census data; enrollment of Asian individuals was similar (5.7%; 95% CI, 5.5%-6.0%). Enrollment of Hispanic or Latino individuals (11.6%; 95% CI, 11.1%-12.0%) was also low even among the limited number of adult trials reporting ethnicity. Adult trials were composed of more female participants (75 325 [56.0%]), but among those reporting age as a percentage, enrollment of participants who were aged 65 years or older was low (12.1%; 95% CI, 12.0%-12.3%). Black or African American participants (10.1%; 95% CI, 9.7%-10.6%) and Hispanic or Latino participants (22.5%; 95% CI, 21.6%-23.4%) were also underrepresented in pediatric trials. Among trials reporting race/ethnicity, 65 (48.5%) did not include American Indian or Alaska Native participants and 81 (60.4%) did not include Hawaiian or Pacific Islander participants.

**Conclusions and Relevance:**

This cross-sectional study found that among US-based vaccine clinical trials, members of racial/ethnic minority groups and older adults were underrepresented, whereas female adults were overrepresented. These findings suggest that diversity enrollment targets should be included for all vaccine trials targeting epidemiologically important infections.

## Key Points

**Question:**

Do vaccine clinical trials equitably represent individuals who identify as members of underrepresented racial/ethnic groups, are women, and are people aged 65 years or older?

**Findings:**

In this cross-sectional study of 230 US-based clinical trials with 219 555 participants, Black or African American, American Indian or Alaska Native, Hispanic or Latino, and older adults were underrepresented and women were overrepresented compared with the US population.

**Meaning:**

The findings suggest that diversity enrollment targets are needed for vaccine trials in the US.

## Teaser

This cross-sectional study examines whether racial and/or ethnic minority, female, and older adults are underrepresented in vaccine clinical trials compared with the US population.

## Introduction

Vaccines are one of the most important public health developments over the past century, leading to global reductions in morbidity and mortality from major infectious diseases.^1,2^ Worldwide production and availability of vaccines have helped to eliminate smallpox and nearly eradicate poliovirus.^3^ Vaccines have been a major focus to address the global severe acute respiratory syndrome coronavirus 2 (SARS-CoV-2) (ie, coronavirus disease 2019 [COVID-19]) pandemic, which is a danger to health care systems and global economies as well as to human life. The US government, in collaboration with industry, has accelerated multiple vaccine candidates through clinical trials with the hope of alleviating the COVID-19 pandemic.^4^

Vaccines are usually only approved for public use after rigorous randomized clinical trials that establish safety and efficacy.^5^ Efforts have been made to increase the inclusion of female adults, older adults, and racially/ethnically diverse participants in clinical trials so that they align with US demographics.^6,7,8^ In vaccine trials, enrollment should target populations at greatest risk for infection, serious morbidity, or mortality. In the case of SARS-CoV-2, data demonstrate disproportionate rates of infection and COVID-19–attributable mortality among older adults and in communities with longstanding social and structural inequities, specifically, Black or African American, American Indian and Alaska Native, Native Hawaiian and Pacific Islander, and Hispanic or Latino individuals.^9,10^ Some minority groups, including but not limited to Black, Latino, Pacific Islander, and Indigenous individuals, have been reported to have more than twice the mortality rate of White people.^10,11^

Historically, clinical trials have lacked equitable inclusion of people identifying as members of racial/ethnic minority groups and female and older individuals.^12^ When people with diverse backgrounds are not adequately represented, treatments shown to be effective in trials may not be generalizable to or effective for all populations.^13^ Furthermore, because of previous experience with exclusion and maltreatment, vaccine hesitancy and lack of trust in the medical establishment may be more prevalent across minority groups, making inclusion even more important.^14^

To enhance enrollment of underrepresented groups, the National Institutes of Health (NIH) Revitalization Act of 1993 mandated appropriate inclusion of women and racial/ethnic minority groups in clinical trials.^15^^,^^16^ However, decades later, inclusion has remained persistently low.^17^ More recently, the US Food and Drug Administration (FDA) developed plans to enhance inclusion of underrepresented groups and to improve reporting of demographic data; the impact of this plan is unclear.^18^

Despite policies aimed at addressing enrollment diversity in clinical trials, to our knowledge, data regarding the inclusion of these groups have not been assessed in vaccine trials. This study assessed whether racial/ethnic minority groups and female and older adults are underrepresented in vaccine clinical trials. We hypothesized that these 3 groups would be underrepresented.

## Methods

This cross-sectional study used data from completed interventional vaccine trials from July 1, 2011, to June 30, 2020, that were registered and reported on ClinicalTrials.gov. On July 17, 2020, the terms *vaccine*, *vaccination*, *immunization*, and *inoculation* were used to identify trials that were registered in the NIH’s ClinicalTrials.gov online repository within the past decade (July 1, 2011, to June 30, 2020) of all human vaccine trials including children only, adults only, or both children and adults and targeting an infection. Trials addressing vaccine immunogenicity or efficacy of preventative vaccines were included; therapeutic vaccines (eg, targeting cancer) were excluded. To be included, trials had to be categorized as completed and have available results. Trials that were completed but without results reported were excluded. Also excluded were studies with unknown status and those that were not yet recruiting, still recruiting, enrolling by invitation, active but not recruiting, suspended, terminated, or withdrawn. Studies based outside the US were excluded, as were studies testing other components of vaccine production, delivery, and participant behavior. Since this cross-sectional study did not involve human participants and used publicly available data, it did not require review and approval by an institutional review board or ethics committee, according to the Fred Hutchinson Cancer Research Center Institutional Review Board. No informed consent was obtained, given that the data used did not contain any identifiable information and participants could not be contacted. This study followed the Strengthening the Reporting of Observational Studies in Epidemiology (STROBE) reporting guideline.

### Data Collection

We collected demographic data, including race/ethnicity, sex, and age. For race/ethnicity, we used the categories used on ClinicalTrials.gov by the Office of Management and Budget Standards for the Classification of Federal Data on Race and Ethnicity.^17,19^ We included the following categories for race: White, Black or African American, Asian, Hawaiian or Pacific Islander, American Indian or Alaska Native, more than 1 race, and other, missing, or unknown. Ethnicity was classified as Hispanic or Latino. Trials were categorized by primary pathogen (eg, viral, bacterial, or other), age (eg, adult, child, or adult and child), and trial phase (eg, phase 1, 2, 3, or 4). For consistency, studies that were characterized as combined phases (eg, phases 1 and 2) were categorized to the higher phase except those that enrolled fewer than 50 participants, which were considered as phase 1 for the purposes of this study; all postlicensure studies were considered phase 4. Month and calendar year for trial start and conclusion were collected to assess reporting changes over the study period. In addition, we collected primary and secondary funding sources for each trial (eg, industry, government). For comparison of these demographic data, we used US population data from the 2011 and 2018 American Community Surveys (ie, US censuses).^20,21^

### Statistical Analysis

For trials reporting demographic information as percentages only, we computed integer counts of participants by multiplying the percentage in each group by the total number of participants analyzed and rounding to the nearest integer. In the event that counts of mutually exclusive minority groups did not add to the total number analyzed in the trial, we adjusted counts among the other, unknown, or missing category so the sum of participants in all minority groups matched the total number of participants analyzed in the trial. To make inference from our sample of vaccine trials to a larger population, we computed 95% CIs for estimates of the percentage in each demographic group using the Wilson method for binomial proportions.^22^ For racial group estimates, we computed simultaneous 95% CIs by applying a Bonferroni correction using a 2-sided α level of .05 divided by 7 to reflect multinomial distribution of race proportions. We compared our estimates with 95% CIs with US population estimates from the 2011 and 2018 censuses, which generally reported a sampling error of ±0.1% for demographic estimates of interest in this study. Analyses were completed using SPSS, version 25.0 (IBM) or SAS, version 9.4 (SAS Institute Inc).

## Results

### Trial Characteristics

**Figure 1.**
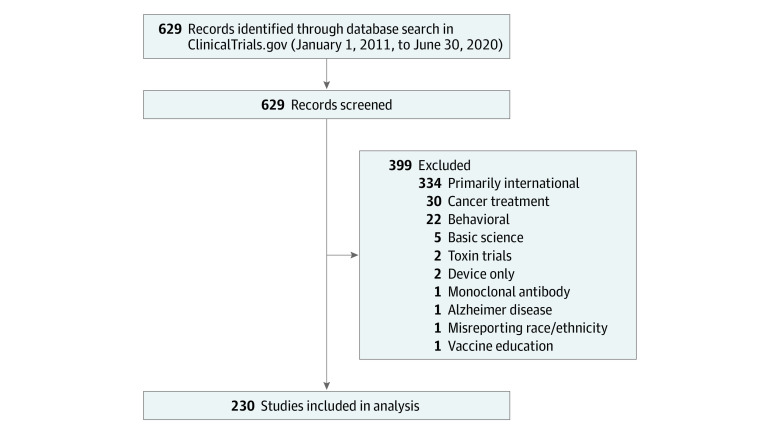
Flowchart of Clinical Trials Included in This Study

A total of 629 studies were identified from the search, of which 399 were excluded (Figure 1). In total, 230 US-based clinical trials with a total of 219 555 participants met the criteria for inclusion (Table 1). Most trials were randomized (180 [78.3%]) and included multiple regions in the US (eFigure 1 in the Supplement); 48 (20.9%) included international testing sites. Viral vaccines were most common (159 [69.1%]), and all trial phases were represented among those analyzed. The percentage of trials that reported age (230 [100%]), sex (230 [100%]), race (134 [58.3%]), and ethnicity (79 [34.3%]) varied (Table 1).

**Table 1.** Characteristics of Registered Vaccine Clinical Trials in ClinicalTrials.gov

Characteristic	No. of trials/No. of trials analyzed (%) (N = 230)
Randomized	180 (78.3)
International sites included	48 (20.9)
Race reported	134 (58.3)
Ethnicity reported	79 (34.3)
Sex reported	230 (100)
Age reported	230 (100)
Aged ≥65 y in inclusion criteria	80 (34.8)
Adults aged ≥65 y enrolled among trials with age ≥65 in inclusion criteria^a^	71 (88.8)
Adults aged ≥65 y enrolled among all adult trials^b^	71 (39.9)
Age category	
Adult	153 (66.1)
Child	52 (22.6)
Both	25 (10.9)
Microorganism	
Viral	159 (69.1)
Bacterial	60 (26.1)
Other^c^	11 (4.8)
Trial phase	
1	29 (12.6)
2	72 (31.3)
3	61 (26.5)
4	68 (29.6)

^a^ Total of 80 trials.

^b^ Total of 178 trials.

^c^ Included fungal, parasitic, bacterial, and viral.

### Race/Ethnicity

Table 2 presents the number and percentage of participants in each demographic group for the subset of 153 adult trials and 52 pediatric trials compared with US census data from 2011 and 2018. Among 91 adult trials reporting race, White participants were overrepresented (77.9%; 95% CI, 77.4%-78.4%), whereas Black or African American participants (10.6%; 95% CI, 10.2%-11.0%) and American Indian and Alaska Native participants (0.4%; 95% CI, 0.3%-0.5%) were underrepresented independent of year of census; Asian participants appeared to be equally represented (5.7%; 95% CI, 5.5%-6.0%) (Table 2). In adult trials reporting ethnicity, Hispanic or Latino participants were underrepresented (11.6%; 95% CI, 11.1%-12.0%; 16.7% of the adult population in 2011 and 18.5% in 2018). Among pediatric trials, Black or African American participants (10.1%; 95% CI, 9.7%-10.6%), Hispanic or Latino participants (22.5%; 95% CI, 21.6%-23.4%), and participants reporting multiple races (1.6%; 95% CI, 1.5%-1.8%) were underrepresented compared with pediatric census data (Table 2).

**Table 2.** Race/Ethnicity and Sex Representation in Registered Vaccine Clinical Trials in ClinicalTrials.gov

Characteristic	Adult trials			Pediatric trials		
	Participants, No. (%) [95% CI] (n = 49 459)^a^	US adult population in 2011, %^b^	US adult population in 2018, %^b^	Participants, No. (%) [95% CI] (n = 32 930)^a^	US child population 2011, %^b^	US child population 2018, %^b^
Race reported						
White	38 543 (77.9) [77.4-78.4]	74.1	76.3	21 261 (64.6) [63.9-65.3]	68.2	66.6
Black or African American included	5246 (10.6) [10.2-11.0]	12.6	13.9	3339 (10.1) [9.7-10.6]	14.3	14.0
Asian included	2832 (5.7) [5.5-6.0]	4.8	5.9	3569 (10.8) [10.4-11.3]	4.4	4.8
American Indian or Alaska Native included	202 (0.4) [0.3-0.5]	0.8	1.3	2290 (7.0) [6.6-7.3]	1.0	1.0
Hawaiian or Pacific Islander included	83 (0.2) [0.1-0.2]	0.2	0.2	118 (0.4) [0.3-0.5]	0.2	0.2
>1 Race	1129 (2.3) [2.1-2.5]	2.8	2.8	538 (1.6) [1.5-1.8]	6.4	6.9
Other, unknown, missing	1424 (2.9) [2.7-3.1]	4.7	NA	1815 (5.5) [5.2-5.9]	5.6	6.4
Ethnicity reported^c^						
Hispanic or Latino	2313 (11.6) [11.1-12.0]	16.7	18.5	1941 (22.5) [21.6-23.4]	23.6	25.4
Sex reported^d^						
Female	75 325 (56.0) [55.7-56.2]	51.5	50.8	29 174 (48.0) [47.6-48.4]	48.8	48.8

Abbreviation: NA, not applicable.

^a^ Wilson 95% CIs are given. For race, simultaneous 95% CIs were calculated using a Bonferroni correction for the 7 racial categories.

^b^ From US census data in the respective year.^20,21^

^c^ Ethnicity was reported for 19 995 adult participants and 8626 pediatric participants.

^d^ Excluding 13 trials that targeted exclusively women from the adult trials. For adult trials, n = 134 570; for pediatric trials, n = 60 767.

**Figure 2.**
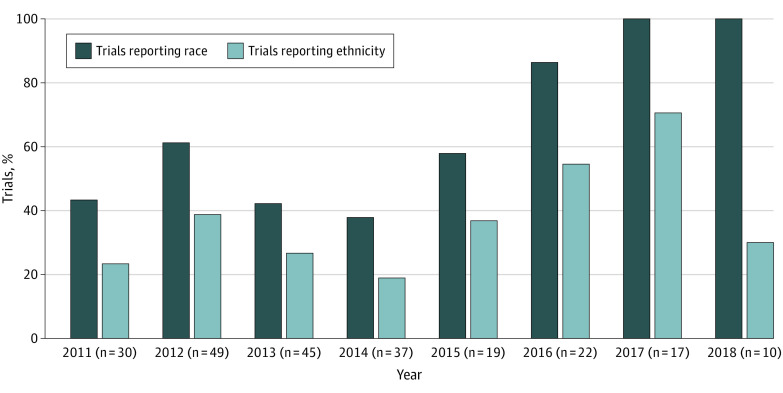
Registered Vaccine Clinical Trials Reporting Race/Ethnicity By Year

Of all 134 trials reporting race, 65 (48.5%) did not include American Indian or Alaska Native participants and 81 (60.4%) did not include Hawaiian or Pacific Islander participants. In contrast, White participants were represented in 133 trials [99.3%] reporting race. There was significant variability in trials reporting race/ethnicity over time. Figure 2 shows the percentage of registered trials reporting race/ethnicity between 2011 and 2018. In 2017 and 2018, 100% of trials reported race, and in 2017, reporting of ethnicity reached its highest percentage (12 of 17 [70.6%]). In most years, fewer than half of the trials reported ethnicity.

**Figure 3.**
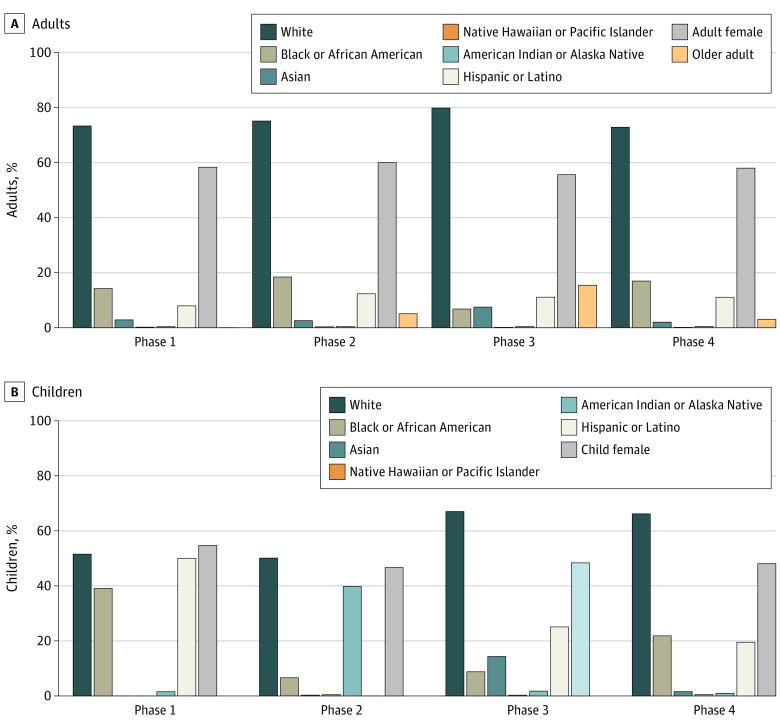
Demographic Representation by Trial Phase A, Percentages for older adults were calculated among all trials reporting age as a percentage.

When analyzed by phase, enrollment of members of racial/ethnic minority groups varied widely (Figure 3). In adult phase 3 trials, 25 684 participants (79.8%) were White and 2187 participants (6.8%) were Black or African American compared with 12.6% to 13.9% reported in the general US population during this period. Similarly, Native Hawaiian and Pacific Islander, American Indian and Alaska Native, and Hispanic or Latino participants were underrepresented in phase 3 trials compared with their representation in the US population. Pediatric trials were more representative of the population when analyzed by phase; however, in pediatric phase 3 trials, 2147 (8.8%) of participants were Black or African American, compared with 14% among the US pediatric population (Figure 3).

### Sex

Overall, female adult participants were overrepresented in vaccine clinical trials. They accounted for 56.0% (95% CI, 55.7%-56.2%) of participants compared with 50.8% to 51.5% of the US population according to census data. When analyzed by phase, female adults remained overrepresented in all trials regardless of phase (Figure 3). In pediatric trials, female participants had nearly equal representation (48.0%; 95% CI, 47.6%-48.4%) compared with the US population (48.8%) (Table 2).

### Age

Of the total sample of 230 trials, 178 recruited adults and 52 recruited only children (Table 1). Of the trials including adults, 80 (45%) had age greater than or equal to 65 years as an inclusion criterion and 71 (39.9%) enrolled trial participants 65 years or older; 5 trials (2.8%) recruited only older adults. Among the 170 studies reporting age as a percentage, 12.1% (95% CI, 12.0%-12.3%) of participants were 65 years or older, in contrast with 16.0% of the 2018 US population representing this age group. When analyzed by phase, older adults were underrepresented across all trial phases, but their greatest participation was in phase 3 trials (Figure 3).

## Discussion

In this cross-sectional study, we hypothesized that individuals who identify as members of minority groups, female adults, and people 65 years or older would be underrepresented in vaccine clinical trials. One of the most important findings was that despite FDA recommendations, many studies were not complying with reporting guidance regarding demographic characteristics of the study population. Black or African American and American Indian or Alaska Native adults were underrepresented, as were Hispanic or Latino adults and older adults. Female adults were overrepresented. Asian and Native Hawaiian or Pacific Islander participants were equally represented in vaccine trials compared with the US population.

The initial NIH Revitalization Act provided the impetus for policies related to the inclusion of women and minority groups in clinical trials for more than a quarter of a century.^16,17^ In 2000, this policy was updated to require inclusion of women and members of minority groups in all “biomedical and behavioral research projects involving human subjects”^8^ unless there was a clear justification for their exclusion. Reporting guidance for trial results were clarified regarding sex, gender, and/or race/ethnicity differences.^16^ In 2001, the NIH updated the policy to align with the Office of Management and Budget guidelines regarding race/ethnicity.^19^ This update provided revised minimum standards of inclusion of sex, gender, and racial/ethnic minority groups in phase 3 clinical trials. The updated policy with diversity amendments stated that investigators needed to collect this information to meet the following definition for clinical research: “Research conducted on human subjects (or material of human origin such as tissues, specimens, and cognitive phenomena) for which an investigator (or colleague) directly interacts with human subjects.”^8^

Nearly 2 decades later, in 2017, the NIH announced an amendment requiring investigators to not only include the specified groups in NIH-defined phase 3 clinical trials but also submit their results to ClinicalTrials.gov after conducting valid analyses based on sex, gender, and race/ethnicity. These analyses entailed an unbiased evaluation of the different groups with appropriate randomization, documentation of different outcomes or measures, and comparison of the intervention effects based on sex, gender, and race/ethnicity. The new rules and regulations were required and applied to all new competing grants awarded from December 2017 onward.^8^ The same year, the NIH updated its existing 1998 policy on the inclusion of children in research to include older individuals, focusing on participants across the life span; exclusion of these age groups requires an explanation.^7^

Despite advancements, equity in clinical trial enrollment remains an issue. In a report examining the frequency in which minorities were the primary focus of National Cancer Institute–sponsored clinical trials, the researchers found, in a search done in 2013 over the previous 2 decades, that less than 2% of these clinical trials had minorities as the primary focus and that the percentage of manuscripts reporting race/ethnicity data ranged from 1.5% to 58%.^17^ Our study contributes additional data to the reported gaps in participant representation; although of importance, the gaps were not large in general.^23,24^ Our study showed that diversity and inclusion challenges remained among vaccine trials because many trials enrolled participants while these updated regulations were already in place. Successful inclusion of underrepresented groups in COVID-19 vaccine trials should help guide future trials.^12,25,26^

We confirmed our first hypothesis that adult participants from minority groups would be underrepresented; however, a large percentage of trials had no data available on minority groups, particularly Hispanic and Latino populations. A lack of reporting is a key finding of our study, and missing data may be important in the context of understanding health disparities, such as social determinants of health (eg, socioeconomic barriers), implicit bias, and an increased burden of comorbidities.^24,27,28,29,30,31,32,33^ For example, neighborhood poverty level has been associated with significant disparities in pneumococcal disease incidence among patients with vaccine and nonvaccine serotypes.^31^ These findings, combined with studies that document lower vaccination rates and increased disease burdens in some of these populations, demonstrate the need to improve minority group enrollment in clinical trials.^34,35,36^

Our findings suggest that NIH policies on reporting of identified groups has increased over time, but a need to focus such policies beyond reporting to representative enrollment remains. Such differences were most apparent in large phase 3 trials in which Black or African American and other underrepresented minority research participants in both adult and pediatric vaccine trials were often underrepresented. Costs and rapid enrollment goals in phase 3 trials, which are primarily funded by industry (eFigure 2 in the Supplement), may be associated with this difference. Our data suggest that investments by federal agencies and industry are needed to ensure that enrollment is fair and equitable. Programs aimed at improving vaccine access for members of minority groups have been shown to be effective in increasing vaccination rates, suggesting that similar support could be used to bolster enrollment numbers.^33^

Improving racial/ethnic diversity in clinical trials is important because enrollment may be associated with vaccination rates in minority groups. Efforts to improve inclusion may help to address vaccine hesitancy, provide education, and counter safety concerns about vaccines by ensuring equitable representation in definitive clinical trials.^37,38,39^ Enhancing enrollment may also address these issues by ensuring early community engagement. In addition, improving the diversity of the health care and clinical trial workforces may further engender trust, ensure culturally appropriate education, and limit language barriers to enhance trial participation and vaccination rates within minority communities.^40^ Future studies are needed to assess whether access to and enrollment in clinical trials will lead to improved vaccination rates in these populations because not all policy changes help improve rates.^41^

Our second hypothesis that female participants would be underrepresented in vaccine clinical trials was disproved. Women have historically been both underrepresented and overrepresented in clinical trials.^42,43,44^ Our data revealed that female adults were overrepresented in trials of all phases. Even when we excluded trials targeting exclusively female individuals, they remained overrepresented. The reasons for this are not clear, and further investigation may be enlightening, perhaps providing guidance for other research areas in which female participants are underrepresented.

Our third hypothesis, that people 65 years or older would be underrepresented, was challenging to assess because of data-reporting issues related to ClinicalTrials.gov. Variance in age reporting allowed us to assess enrollment of older adults in less than one-half of all trials. Considering trials that allowed assessment of this population and excluding those targeting only older adults, adults 65 years or older were less frequently enrolled in trials than expected according to their representation in the US population based on census data.^45^ This finding is not unexpected because many clinical vaccine trials target primary end points of seroconversion, immunogenicity, and/or efficacy and older adults have altered immunity with aging and less robust responses to conventional vaccines.^46,47^

Our data suggest a need to enroll more older adults, particularly for vaccines targeting infections, such as SARS-CoV-2 infection, that are known to be associated with excess morbidity and mortality among older adults.^48,49,50,51^ Although we focused on the representation of older adults, of note, the data challenges mentioned previously also apply to pediatric trials. The incomplete and varied reporting among specific age groups of children limited our ability to assess inclusion across the life span.^19^

### Limitations

This study has limitations. We examined 230 US-based trials with 219 555 participants; however, a lack of compliance with reporting data on the ClinicalTrials.gov website is both an important finding and a limitation. Despite clear grouping labels, data from ClinicalTrials.gov are heterogeneous. For example, although 100% of trials reported some age information, the registry lacks age-reporting standards. Age was reported variably by category, mean, range, and/or percentage older than 65 years, resulting in difficulty addressing age disparities. Most trials reported race/ethnicity using standardized categories, but some used customized race reporting, and we had to deduce racial makeup or eliminate trials entirely because of insufficient data. Furthermore, we relied on 2 US census estimates for population norms for demographic categories because the years when these data were reported spanned the study period. Evaluation of phase 3 trial enrollment may have been biased toward less reporting on race/ethnicity because most completed trials were begun before FDA regulations for reporting were established. ClinicalTrials.gov reports sex data only, which restricted our ability to analyze representation of gender identity minority groups in trials. Similarly, we were unable to analyze representation with regard to sexual orientation because of lack of data reporting. We were unable to account for database inaccuracies in ClinicalTrials.gov.

## Conclusions

In this cross-sectional study, among adult participants in US vaccine clinical trials, the racial/ethnic minority groups that we studied and people 65 years or older were underrepresented, whereas female adults were overrepresented. Populations with an increased infectious disease burden should be equitably included in trials of vaccines for COVID-19 and other vaccines.

## Notes

Supplement. **eFigure 1.** Geographic Distribution of Vaccine Trials **eFigure 2.** Funding Entities of Vaccine Clinical Trials

## References

[1] The Lancet Infectious Diseases The imperative of vaccination. Lancet Infect Dis. 2017;17(11):1099. doi:10.1016/S1473-3099(17)30590-X 29115253

[2] Orenstein WA, Ahmed R Simply put: vaccination saves lives. Proc Natl Acad Sci U S A. 2017;114(16):4031-4033. doi:10.1073/pnas.1704507114 28396427 PMC5402432

[3] Lickness JS, Gardner T, Diop OM, Surveillance to track progress toward polio eradication—worldwide, 2018-2019. MMWR Morb Mortal Wkly Rep. 2020;69(20):623-629. doi:10.15585/mmwr.mm6920a3 32437342

[4] O’Callaghan KP, Blatz AM, Offit PA Developing a SARS-CoV-2 vaccine at warp speed. JAMA. 2020;324(5):437-438. doi:10.1001/jama.2020.12190 32628244

[5] Grady C, Shah S, Miller F, So much at stake: ethical tradeoffs in accelerating SARSCoV-2 vaccine development. Vaccine. 2020;38(41):6381-6387. doi:10.1016/j.vaccine.2020.08.017 32826103 PMC7418641

[6] Chastain DB, Osae SP, Henao-Martínez AF, Franco-Paredes C, Chastain JS, Young HN Racial disproportionality in Covid clinical trials. N Engl J Med. 2020;383(9):e59. doi:10.1056/NEJMp2021971 32780573

[7] National Institutes of Health, US Department of Health and Human Services Inclusion across the lifespan. Updated July 10, 2019. Accessed September 11, 2020. https://grants.nih.gov/policy/inclusion/lifespan.htm

[8] US Department of Health & Human Services. National Institutes of Health NIH policy and guidelines on the inclusion of women and minorities as subjects in clinical research. Updated December 6, 2017. Accessed September 11, 2020. https://grants.nih.gov/policy/inclusion/women-and-minorities/guidelines.htm

[9] Mackey K, Ayers CK, Kondo KK, Racial and ethnic disparities in COVID-19-related infections, hospitalizations, and deaths: a systematic review. Ann Intern Med. Published online December 1, 2020. doi:10.7326/M20-6306 33253040 PMC7772883

[10] Tai DBG, Shah A, Doubeni CA, Sia IG, Wieland ML The disproportionate impact of COVID-19 on racial and ethnic minorities in the United States. Clin Infect Dis. 2020;ciaa815. Published online June 20, 2020. doi:10.1093/cid/ciaa815 32562416 PMC7337626

[11] APM Research Lab The color of coronavirus: COVID-19 deaths by race and ethnicity in the U.S. Accessed January 15, 2021. https://www.apmresearchlab.org/covid/deaths-by-race

[12] Loree JM, Anand S, Dasari A, Disparity of race reporting and representation in clinical trials leading to cancer drug approvals from 2008 to 2018. *JAMA Oncol* 2019;5(10):e191870. doi:10.1001/jamaoncol.2019.1870 PMC6696743 31415071

[13] Ramamoorthy A, Pacanowski MA, Bull J, Zhang L Racial/ethnic differences in drug disposition and response: review of recently approved drugs. Clin Pharmacol Ther. 2015;97(3):263-273. doi:10.1002/cpt.61 25669658

[14] Corbie-Smith G, Thomas SB, St George DMM Distrust, race, and research. Arch Intern Med. 2002;162(21):2458-2463. doi:10.1001/archinte.162.21.2458 12437405

[15] Knepper TC, McLeod HL When will clinical trials finally reflect diversity? Nature. 2018;557(7704):157-159. doi:10.1038/d41586-018-05049-5 29743700

[16] Freedman LS, Simon R, Foulkes MA, Inclusion of women and minorities in clinical trials and the NIH Revitalization Act of 1993—the perspective of NIH clinical trialists. *Control Clin Trials* 1995;16(5):277-285; discussion 286-279, 293-309. 10.1016/0197-2456(95)00048-8 8582146

[17] Chen MS Jr, Lara PN, Dang JHT, Paterniti DA, Kelly K Twenty years post–NIH Revitalization Act: enhancing minority participation in clinical trials (EMPaCT): laying the groundwork for improving minority clinical trial accrual: renewing the case for enhancing minority participation in cancer clinical trials. *Cancer* 2014;120 (suppl 7):1091-1096. 10.1002/cncr.28575 PMC3980490 24643646

[18] US Food and Drug Administration FDA action plan to enhance the collection and availability of demographic subgroup data. Published August 2014. Accessed September 15, 2020. https://www.fda.gov/media/89307/download

[19] National Institutes of Health; US Office of Budget and Management Revisions to the standards for the classification of federal data on race and ethnicity. Published 2020. Accessed September 8, 2020. https://orwh.od.nih.gov/toolkit/other-relevant-federal-policies/OMB-standards

[20] US Census Bureau ACS demographic and housing estimates: survey program: American Community Survey; 2011. Accessed September 11, 2020. https://data.census.gov/cedsci/table?q=Race&t=Race%20and%20Ethnicity&tid=ACSDP5Y2011.DP05&hidePreview=true

[21] US Census Bureau QuickFacts: population estimates. Published July 1, 2019. Accessed March 16, 2020. https://www.census.gov/quickfacts/fact/table/US/PST045219

[22] Brown LD, Cai TT, DasGupta A Interval estimation for a binomial proportion. Stat Sci. 2001;16(2):101-117. doi:10.1214/ss/1009213286

[23] Niranjan SJ, Martin MY, Fouad MN, Bias and stereotyping among research and clinical professionals: perspectives on minority recruitment for oncology clinical trials. Cancer. 2020;126(9):1958-1968. doi:10.1002/cncr.32755 32147815

[24] Hamel LM, Penner LA, Albrecht TL, Heath E, Gwede CK, Eggly S Barriers to clinical trial enrollment in racial and ethnic minority patients with cancer. Cancer Control. 2016;23(4):327-337. doi:10.1177/107327481602300404 27842322 PMC5131730

[25] US Food and Drug Administration Moderna COVID-19 vaccine. Presented at: Vaccines and Related Biological Products Advisory Committee Meeting; December 17, 2020 Accessed December 19, 2020. https://www.fda.gov/media/144434/download

[26] Polack FP, Thomas SJ, Kitchin N, ; C4591001 Clinical Trial Group Safety and efficacy of the BNT162b2 mRNA COVID-19 vaccine. N Engl J Med. 2020; 383(27):2603-2615. doi:10.1056/NEJMoa2034577 33301246 PMC7745181

[27] Whittle RS, Diaz-Artiles A An ecological study of socioeconomic predictors in detection of COVID-19 cases across neighborhoods in New York City. BMC Med. 2020;18(1):271. doi:10.1186/s12916-020-01731-6 32883276 PMC7471585

[28] Mahmoudi E, Jensen GA Diverging racial and ethnic disparities in access to physician care: comparing 2000 and 2007. Med Care. 2012;50(4):327-334. doi:10.1097/MLR.0b013e318245a111 22388557

[29] Hall WJ, Chapman MV, Lee KM, Implicit racial/ethnic bias among health care professionals and its influence on health care outcomes: a systematic review. Am J Public Health. 2015;105(12):e60-e76. doi:10.2105/AJPH.2015.302903 26469668 PMC4638275

[30] Molina Y, Silva A, Rauscher GH Racial/ethnic disparities in time to a breast cancer diagnosis: the mediating effects of health care facility factors. Med Care. 2015;53(10):872-878. doi:10.1097/MLR.0000000000000417 26366519 PMC4570266

[31] Raman R, Brennan J, Ndi D, Marked reduction of socioeconomic and racial disparities in invasive pneumococcal disease associated with conjugate pneumococcal vaccines. *J Infect Dis* Published online August 11, 2020. doi:10.1093/infdis/jiaa515 32780860

[32] Millett GA, Jones AT, Benkeser D, Assessing differential impacts of COVID-19 on black communities. Ann Epidemiol. 2020;47:37-44. doi:10.1016/j.annepidem.2020.05.003 32419766 PMC7224670

[33] Poteat T, Millett GA, Nelson LE, Beyrer C Understanding COVID-19 risks and vulnerabilities among black communities in America: the lethal force of syndemics. Ann Epidemiol. 2020;47:1-3. doi:10.1016/j.annepidem.2020.05.004 32419765 PMC7224650

[34] Hirth J, McGrath CJ, Kuo YF, Rupp RE, Starkey JM, Berenson AB Impact of human papillomavirus vaccination on racial/ethnic disparities in vaccine-type human papillomavirus prevalence among 14-26 year old females in the U.S. Vaccine. 2018;36(50):7682-7688. doi:10.1016/j.vaccine.2018.10.075 30377066 PMC6289515

[35] Lu D, Qiao Y, Brown NE, Wang J Racial and ethnic disparities in influenza vaccination among adults with chronic medical conditions vary by age in the United States. PLoS One. 2017;12(1):e0169679-e0169679. doi:10.1371/journal.pone.0169679 28081234 PMC5231366

[36] Winter K, Harriman K Risk markers for pertussis among infants <4 months of age: understanding the Hispanic disparity. Pediatr Infect Dis J. 2018;37(2):126-131. doi:10.1097/INF.0000000000001707 28777209

[37] Walker AT, Smith PJ, Kolasa M; Centers for Disease Control and Prevention (CDC) Reduction of racial/ethnic disparities in vaccination coverage, 1995-2011. MMWR Suppl. 2014;63(1):7-12. 24743661

[38] Moran MB, Frank LB, Chatterjee JS, Murphy ST, Baezconde-Garbanati L Information scanning and vaccine safety concerns among African American, Mexican American, and non–Hispanic White women. Patient Educ Couns. 2016;99(1):147-153. doi:10.1016/j.pec.2015.08.016 26321294 PMC4691412

[39] Quinn SC, Jamison A, An J, Freimuth VS, Hancock GR, Musa D Breaking down the monolith: understanding flu vaccine uptake among African Americans. SSM Popul Health. 2017;4:25-36. doi:10.1016/j.ssmph.2017.11.003 29349270 PMC5769118

[40] Freimuth VS, Jamison AM, An J, Hancock GR, Quinn SC Determinants of trust in the flu vaccine for African Americans and Whites. Soc Sci Med. 2017;193:70-79. doi:10.1016/j.socscimed.2017.10.001 29028558 PMC5706780

[41] Yue D, Rasmussen PW, Ponce NA Racial/ethnic differential effects of Medicaid expansion on health care access. Health Serv Res. 2018;53(5):3640-3656. doi:10.1111/1475-6773.12834 29468669 PMC6153163

[42] Khan SU, Khan MZ, Raghu Subramanian C, Participation of women and older participants in randomized clinical trials of lipid-lowering therapies: a systematic review. JAMA Netw Open. 2020;3(5):e205202. doi:10.1001/jamanetworkopen.2020.5202 32437574 PMC7243092

[43] Duma N, Vera Aguilera J, Paludo J, Representation of minorities and women in oncology clinical trials: review of the past 14 years. J Oncol Pract. 2018;14(1):e1-e10. doi:10.1200/JOP.2017.025288 29099678

[44] Liu KA, Mager NAD Women’s involvement in clinical trials: historical perspective and future implications. Pharm Pract (Granada). 2016;14(1):708. doi:10.18549/PharmPract.2016.01.708 27011778 PMC4800017

[45] DiazGranados CA, Dunning AJ, Kimmel M, Efficacy of high-dose versus standard-dose influenza vaccine in older adults. N Engl J Med. 2014;371(7):635-645. doi:10.1056/NEJMoa1315727 25119609

[46] Whitaker JA, von Itzstein MS, Poland GA Strategies to maximize influenza vaccine impact in older adults. Vaccine. 2018;36(40):5940-5948. doi:10.1016/j.vaccine.2018.08.040 30153995

[47] Burke M, Rowe T Vaccinations in older adults. Clin Geriatr Med. 2018;34(1):131-143. doi:10.1016/j.cger.2017.08.006 29129213

[48] Gupta S, Hayek SS, Wang W, ; STOP-COVID Investigators Factors associated with death in critically ill patients with coronavirus disease 2019 in the US. JAMA Intern Med. 2020;180(11):1-12. doi:10.1001/jamainternmed.2020.3596 32667668 PMC7364338

[49] Pilishvili T, Bennett NM Pneumococcal disease prevention among adults: strategies for the use of pneumococcal vaccines. Vaccine. 2015;33(suppl 4):D60-D65. doi:10.1016/j.vaccine.2015.05.102 26116257

[50] Liu BC, McIntyre P, Kaldor JM, Quinn HE, Ridda I, Banks E Pertussis in older adults: prospective study of risk factors and morbidity. Clin Infect Dis. 2012;55(11):1450-1456. doi:10.1093/cid/cis627 22806592

[51] Czaja CA, Miller L, Alden N, Age-related differences in hospitalization rates, clinical presentation, and outcomes among older adults hospitalized with influenza—U.S. Influenza Hospitalization Surveillance Network (FluSurv-NET). Open Forum Infect Dis. 2019;6(7):ofz225. doi:10.1093/ofid/ofz225 31363771 PMC6602897

