# Supplementary material for: Assessment of the Inclusion of Racial/Ethnic Minority, Female, and Older Individuals in Vaccine Clinical Trials
Source: JAMA Netw Open. 2021 Feb 19;4(2):e2037640. doi: 10.1001/jamanetworkopen.2020.37640 (PMC7896193; doi:10.1001/jamanetworkopen.2020.37640)
Supplement: Supplement. — eFigure 1. Geographic Distribution of Vaccine Trials eFigure 2. Funding Entities of Vaccine Clinical Trials [file jamanetwopen-e2037640-s001.pdf]

Supplementary Online Content

Flores LE, Frontera WR, Andrasik MP, et al. Assessment of the inclusion of racial/ethnic minority, female, and older individuals in vaccine clinical trials. *JAMA Netw Open*. 2021;4(2):e2037640. doi:10.1001/jamanetworkopen.2020.37640

eFigure 1. Geographic Distribution of Vaccine Trials

eFigure 2. Funding Entities of Vaccine Clinical Trials

This supplementary material has been provided by the authors to give readers additional information about their work.

© 2021 Flores LE et al. *JAMA Network Open*.

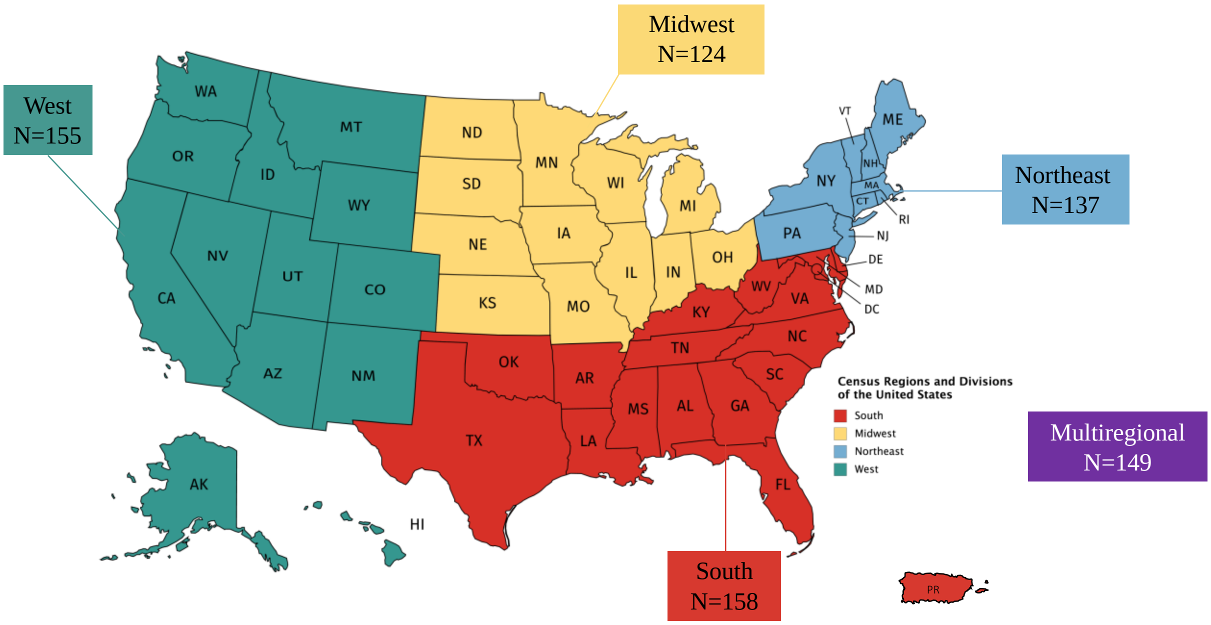

eFigure 1. Geographic Distribution of Vaccine Trials

Legend: This figure demonstrates the distribution of trial sites in the United States as determined by standard census region. Many clinical trials took place at two or more sites, bringing the total to greater than 230. Those with one or two sites were documented within the map. Multiregional represents trials with two sites or greater.

© 2021 Flores LE et al. *JAMA Network Open*.

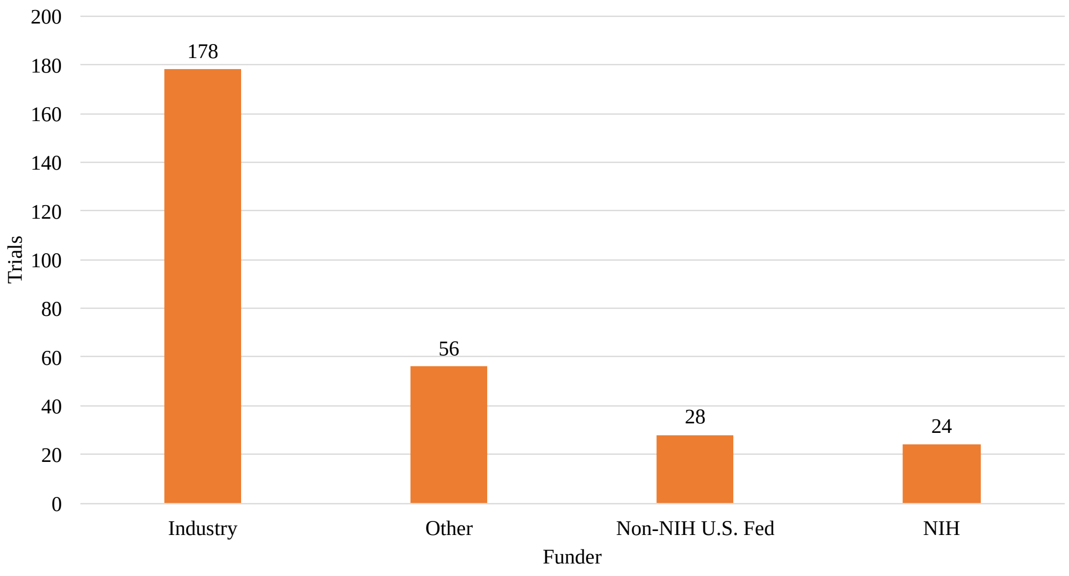

eFigure 2. Funding Entities of Vaccine Clinical Trials
Legend: This figure demonstrates funding entities of vaccine clinical trials. Many trials had more than one funder, bringing the total to greater than 230. Non-NIH U.S. Fed Includes United States Department of Defense, Walter Reed Army Institute of Research (WRAIR) among others.

© 2021 Flores LE et al. *JAMA Network Open*.
